# Genetic Diversity, Population Structure and Subset Development in a *Sesbania sesban* Collection

**DOI:** 10.3390/plants12010013

**Published:** 2022-12-20

**Authors:** Alemayehu Teressa Negawo, Habib Olumide Akinmade, Meki S. Muktar, Ermias Habte, Yilikal Assefa, Alice Muchugi, Alieu M. Sartie, Chris S. Jones

**Affiliations:** 1 Feed and Forage Development, International Livestock Research Institute, Addis Ababa P.O. Box 5689, Ethiopia; 2 Forage Breeding and Genetics, Agronomy Department, University of Florida, Gainesville, FL 32611, USA; 3 The Pacific Community (SPC), Private Mail Bag, Suva, Fiji; 4 Feed and Forage Development, International Livestock Research Institute, Nairobi 00100, Kenya

**Keywords:** DArTSeq, genetic diversity, *Sesbania sesban*, subset

## Abstract

*Sesbania sesban* (L.) Merr. is a multipurpose legume tree grown primarily for fodder and forage in the tropical and subtropical world. In this study, the *Sesbania sesban* collection maintained in the International Livestock Research Institute (ILRI) forage Genebank was studied using genome-wide markers generated on the DArTseq platform. Genotyping produced 84,673 and 60,626 SNP and SilicoDArT markers with a mean polymorphic information content of 0.153 and 0.123, respectively. From the generated markers, 7587 and 15,031 highly informative SNP and SilicoDArT markers, respectively, were filtered and used for genetic diversity analysis and subset development. Analysis of molecular variance (AMOVA) revealed higher variability ‘within’ (52.73% for SNP markers and 67.36% for SilicoDArT markers) than ‘between’ accessions. Hierarchical cluster analysis showed the presence of four main clusters in the collection. Mantel correlation analysis showed a lack of relationship between genetic variation of the germplasm and their geographical origin. A representative subset of 34 accessions containing germplasm from diverse origins and agro-ecologies was developed using SNP markers. The genetic diversity information generated in this study could be used for marker-assisted screening for stress tolerance, gap analysis and identification and acquisition of new distinct genotype(s) to broaden the genetic basis of the collection for future improvement programs to develop high-yielding, stress-tolerant varieties for enhancing food and environmental security in crop–livestock-based production systems.

## 1. Introduction

Sustainable livestock production requires the year-round availability of feed resources. Among the feed resources, forage crops play a key role in helping to ensure that livestock receive a balanced diet in the smallholder mixed farming systems of the tropics and subtropics. *Sesbania sesban* (L.) Merr. is a fast-growing short-lived perennial forage tree in the Leguminosae family [1]. It is native to Africa and Asia [2] and is widely distributed throughout the tropical regions [1,2,3]. It is a diploid species with a basic chromosome number of x = 6 [2,4,5] and is a primarily outcrossing species [2] with 40–100% reported outcrossing levels due to its floral structure and pollen-shedding behaviour [6].

*Sesbania sesban* is a multipurpose legume tree primarily cultivated for forage in tropical and subtropical regions of the world [2,3,7], can produce up to 20 t DM/ha/year under favourable growing conditions, and is a cheap and good source of protein-rich forage for livestock [1,2]. It is also used as an intercrop to provide shade in coffee, tea and cocoa farms, and its leaves can be used as soap and medicine, while young stems can be used to make fibre [3]. The leaves, flowers and seeds can be eaten by people [1]. It can be grown along borders to provide a windbreak, fences and firewood for smallholder farmers [2,3]. It comprises important agronomic features that include the ability to withstand waterlogging and tolerance of moisture stress, soil acidity, alkalinity and salinity [2,3]. Its roots form symbiotic associations with Rhizobium soil bacteria and fix atmospheric nitrogen that improves soil fertility [8] and increases the availability of organic nitrogen to the neighbouring crops, thus playing an important role in improving productivity [2]. The plants provide green manure and mulch of high-nutrient content, together with nitrogen from the nodules that it contributes to soil fertility management, as well as weed control [2,8]. In general, given its multipurpose values, *Sesbania sesban* is one of the most commonly used tree species in agroforestry systems [8]. 

Research and development on the improvement of multipurpose forages like *Sesbania sesban* is crucial, especially in the context of smallholder farmers in the tropical and subtropical regions where feed resources are limited in terms of quality, quantity and year-round availability, and the multiple use of a single resource is highly valued. Developing adaptable and stress-tolerant crop varieties through improvement programmes and promoting the wider adoption and use of such crops requires an in-depth knowledge and understanding of the diversity of the crop species. The International Livestock Research Institute (ILRI) forage genebank holds germplasm of *Sesbania sesban* collected from different countries from around the world. However, except for limited characterisation studies, the collection has not been extensively evaluated for genetic diversity and agronomic performance under different agro-ecologies to select and develop improved lines. Low-density markers, including randomly amplified polymorphic DNA (RAPD) and inter-simple sequence repeat (ISSR) markers, have been used to study the genetic relationship between *Sesbania sesban* accessions [5,9]. Variation in yield performance and insect resistance of accessions from the collection have also been reported [10,11]. More recently, although not yet used for diversity analysis, next-generation sequence-based expressed sequence tag-simple sequence repeat (EST-SSR) markers have been developed by transcriptome sequencing [12]. Like many other forage crops, genomic studies and the application of modern molecular tools such as next-generation sequence-based development of high throughput markers and discovery of genomic regions of interest are lacking in *Sesbania sesban*. Hence, in the current study, we generated a large number of genome-wide molecular markers (SNP and SilicoDArT (presence/absence)) based on next-generation sequencing and studied the genetic diversity of the *Sesbania sesban* collection held in the ILRI forage genebank. The markers were generated on DArTSep platform that combines restriction digestion genome complexity reduction and next-generation sequencing, as described elsewhere [13]. We also used the generated markers to develop a representative subset containing 20% of the collection.

## 2. Results

### 2.1. Informativeness and Diversity of the DArTseq Markers

**Figure 1:**
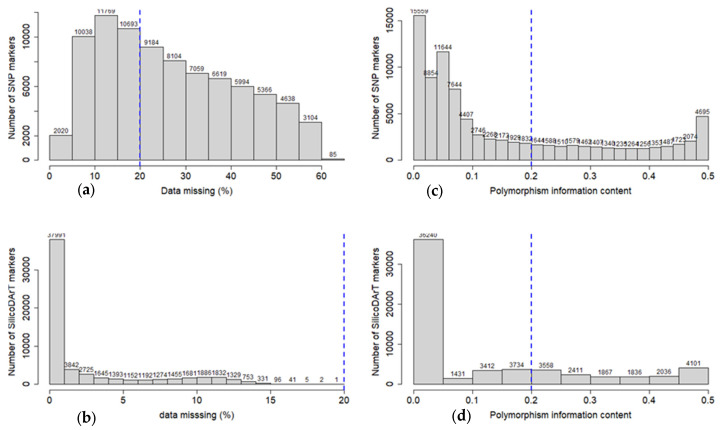
Number of DArTSeq markers by missing data (%) and PIC values for SNP (**a**,**c**) and SilicoDArT markers (**b**,**d**), respectively. The dotted blue vertical line indicated the threshold value for filtered markers.

Genotyping data of 84,673 and 60,626 SNP and SilicoDArT markers, respectively, were generated for 171 *Sesbania sesban* accessions. The missing data percentage ranged from zero to 65% and zero to 26% for SNP and SilicoDArT markers, respectively (Figure 1a,b). The PIC value of the markers ranged from 0.0 to 0.5 (Figure 1c,d) with an average of 0.153 and 0.123 for SNP and SilicoDArT markers, respectively. In general, the genotyping generated enough informative markers (PIC ≥ 0.2, missing data percentage ≤ 20%) to select for downstream genetic diversity and population structure analysis.

**Figure 2:**
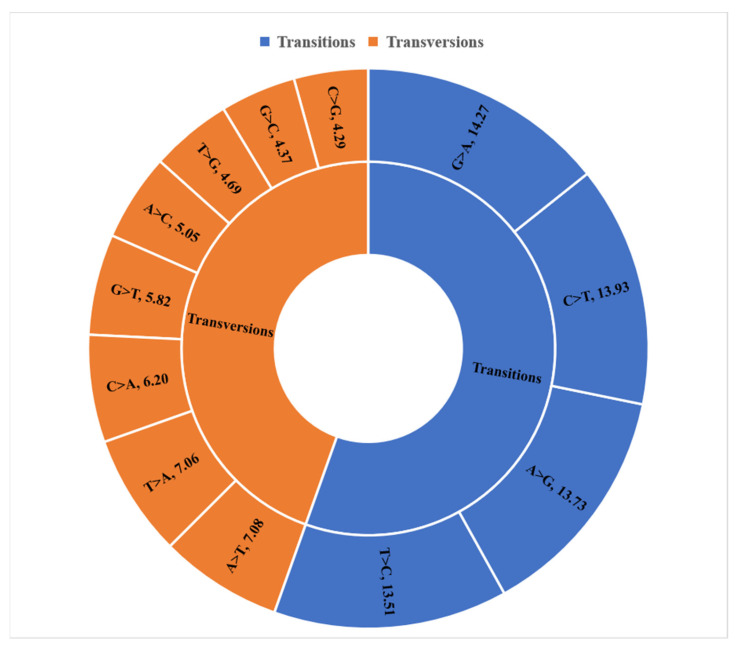
Proportion of SNP markers by transition and transversion polymorphisms.

The sequence length of the markers ranged from 26 to 69 bases with a mean value of 66 bases for both marker types. Over 85.73% of the SNP and 81.11% of the SilicoDArT markers had a fragment length of 69 bases. The types of variation (transitions versus transversions) were also analysed for the SNP markers (Figure 2). Approximately 55.4% of the variation was due to transition polymorphisms while 44.6% of the variation was due to transversions. The proportions of variation due to the different transitions were 15.51% C/T, 14.27% G/A, 13.51% T/C and 13.73% A/G. Similarly, the contribution of the different transversions ranged from 4.29% for C/G to 7.08% for A/T. 

### 2.2. Mapping and Genome-Wide Distribution of the DArTSeq Markers

Sequence fragments of 17.99% and 9.04% of the generated SNP and SilicoDArT markers, respectively, were mapped to the transcriptome sequence of *Sesbania sesban* [12]. In an effort to select markers with known genome position for downstream analyses, the reference genomes of *Medicago truncatula* [14], *Lotus japonicus* [15] and *Pisum sativum* [16] were selected on the basis of their phylogenetic relationship with *Sesbania sesban* and used to map the generated markers (Table 1, Supplementary Figure S1). However, only a small proportion of the generated markers mapped on the reference genomes. Among the reference genomes, the largest number of markers (4.39%) mapped on *Lotus japonicus* while the smallest number of markers (1.43%) mapped on *Medicago truncatula*.

The genome-wide marker density plot showed that the highest number of markers per chromosome mapped on *Lotus japonicus,* with the number of markers per chromosome ranging from 520 to 782 for SNP and 141 to 257 for SilicoDArT markers. This was followed by *Pisum sativum,* with the greatest number of markers per chromosome, ranging from 378 to 559 for SNP and 119 to 257 for SilicoDArT markers. For *Medicago truncatula,* the number of markers per chromosome ranged from 121 to 550 for SNP and 42 to 173 for SilicoDArT markers. The highest and lowest number of markers per chromosome were mapped on chromosome 1 of *Lotus japonicus* and chromosome 6 of *Medicago truncatula*, respectively (see Supplementary Figure S1). 

### 2.3. Between and Within Accession Genetic Diversity

**Figure 3:**
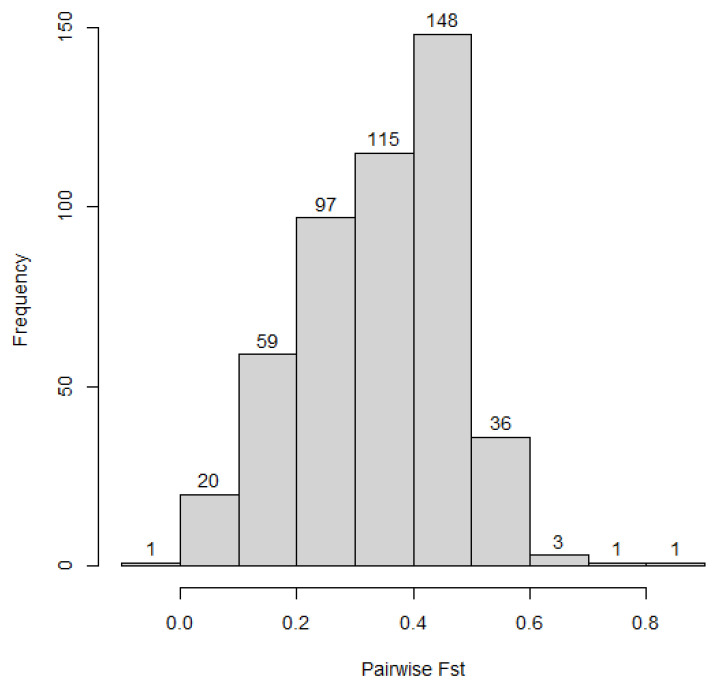
Accessions’ pairwise Fst value.

Table 2 shows the AMOVA result for genetic diversity between and within accessions. The result showed a significant (*p* value = 0.01) contribution of the between accessions’ variation to the total diversity in the collection. However, the within accessions’ variation contributed a larger proportion of the total diversity. The accessions’ pairwise Fst value, using SNP markers, ranged from −0.006 to 0.854 with an average of 0.344 (Figure 3, Supplementary Figure S2). 

### 2.4. Genetic Diversity and Population Structure Detected in the Collection

**Figure 4:**
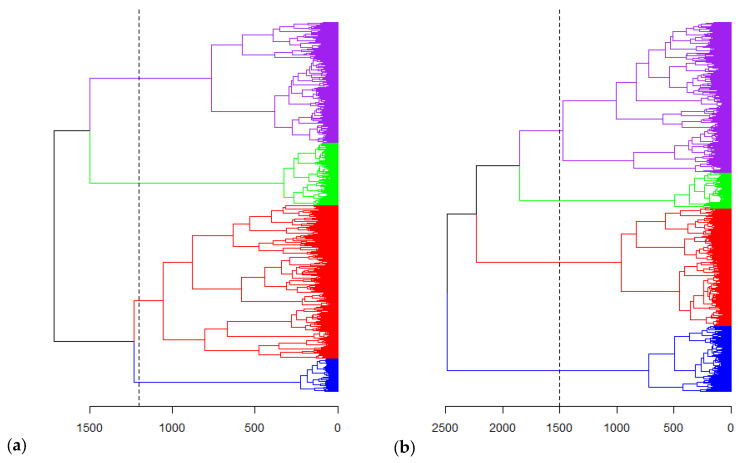
Hierarchical clustering of *Sesbania sesban* accessions by (**a**) SNP and (**b**) SilicoDArT markers.

**Figure 5:**
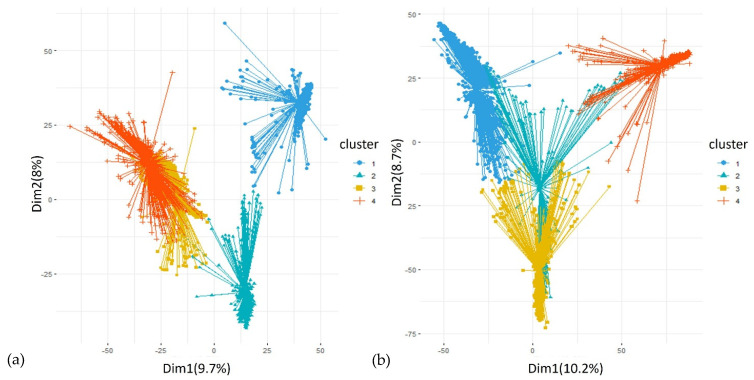
Cluster plots showing the four main clusters using (**a**) SNP and (**b**) SilicoDArT markers.

**Figure 6:**
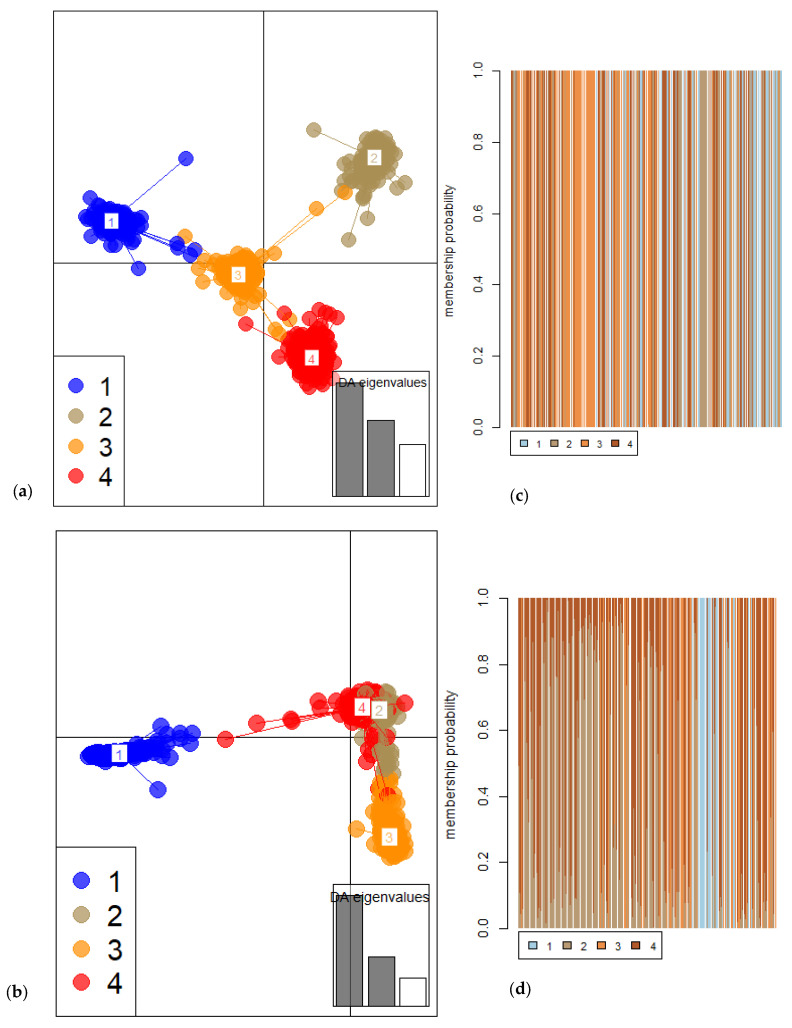
Discriminant analysis of principal components (DAPC) of the *Sesbania sesban* collection. Discriminant analysis by (**a**) SNP and (**b**) SilicoDArT markers. The cluster membership of individual sample inferred from discriminant analysis by (**c**) SNP and (**d**) SilicoDArT markers.

**Figure 7:**
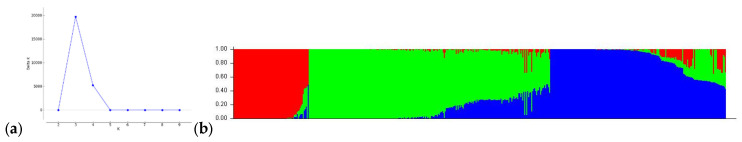
Population structure analysis of the *Sesbania sesban* collection: (**a**) the delta K showing the highest peak at K = 3 suggesting the presence of three subpopulations and (**b**) a bar plot based on the admixture model in STRUCTURE for K = 3.

To analyse the genetic relationship between the accessions, genetic distances were calculated based on the Euclidean method and used for hierarchical clustering. The mantel correlation analysis showed a positive correlation between the genetic distances calculated from SNP and SilicoDArT markers (r = 0.6375, *p*-value = 0.0001). Figure 4 shows the hierarchical clustering of the collection. Using both the SNP and SilicoDArT markers, the collection was assembled into four main groups, with further subgrouping into smaller groups. The hierarchical clusters generated from the SNP and SilicoDArT markers had a cophenetic correlation coefficient of 90.83%**.** Figure 5 shows the cluster plots of the accessions showing the four main groups. The result of the DAPC showing cluster membership of individual sample in the four clusters is shown in Figure 6. For the SNP markers, the first and second dimensions of the cluster plot explained 16.5% of the total genetic variation. Similarly, structure analysis was used to analyse the presence of subpopulations in the collection. Accordingly, the highest peak for delta K was observed at K = 3, indicating the presence of three subpopulations in the collection (Figure 7). 

Table 3 shows the AMOVA result for clusters and subpopulations inferred based on hierarchical clustering and structure analysis, respectively. The results show that the largest proportion (64.28% and 73.39% using SNP and SilicoDArT markers, respectively) of the total variation was contributed by the within cluster variation. The contribution of between clusters’ variation to the total genetic variation was 35.72% and 26.61% using SNP and SilicoDArT markers, respectively. 

**Figure 8:**
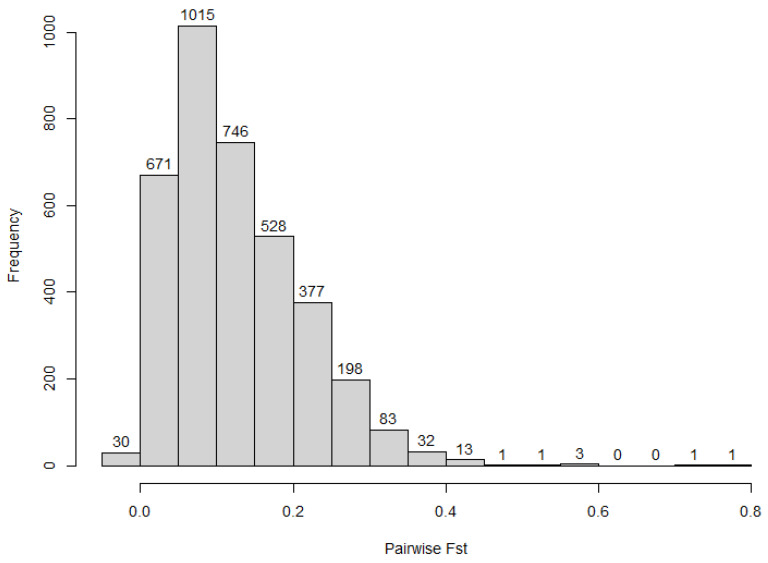
Population pairwise Fst value.

Passport data shows that 161 accessions in the collection were obtained from 25 countries. Of these, coordinate (latitude and longitude) information is available for 136 accessions. The coordinate information was converted to geographical distances using the distm() function of the R package geosphere [17] and was then used for Mantel correlation analysis to assess the relationship between the geographical and genetic distances. The results showed non-significant correlation between the geographical and genetic distances (r = 0.097, *p*-value = 0.055 for SNP and r= 0.06158, *p*-value= 0.123 for SilicoDArT markers). We also conducted analysis of molecular variance for the population according to their geographical origin and assessed how the genetic differentiation is related to the geographical origin of the accessions. The results revealed the within population diversity contributed a large proportion of the total diversity (Table 4). Despite a small proportion, the variation between populations collected from different countries of origin was significant, indicating the uniqueness of the accessions from the different countries. Population pairwise Fst value ranged from −0.007 to 0.782 with a mean of 0.126 using SNP markers (Figure 8, Supplementary Figure S3).

### 2.5. Subset Development

The filtered informative SNP markers were used to develop a representative subset containing 20% of the collection. Within accession identity by descent (IBD) was calculated using the R package SNPRelate [18], and samples with a kinship of ≥0.30 were retained for subset development. Then, a representative sample per accession was selected and used for subset establishment. The developed subset contained 34 accessions collected from diverse geographical origins (Table 5), including: 12 accessions from Tanzania; six from Ethiopia; three from Kenya; five from Malawi; one each from Central African Republic, Namibia, Uganda, India, Zambia, Zimbabwe and Mexico; and one accession of unknown origin. AMOVA was used to assess the representativeness of the subset, and the result revealed the about 96∓99% of the genetic variation was captured by the developed subset (Table 6).

## 3. Discussion

### 3.1. Genotyping and Informativeness of DArTSeq Markers 

Genomic tools such as next-generation sequencing and bioinformatics packages have advanced the genetic studies of many orphan crops, and their application in tropical forage crops has increased in recent years. However, so far *Sesbania sesban* has not been studied using genome-wide high-throughput markers generated by next-generation sequencing. In this study, we investigated the genetic diversity in a *Sesbania sesban* collection held in the ILRI forage genebank using the genotyping-by-sequencing (GBS) approach of the DArTSeq platform [13]. A large number of SNP and SilicoDArT markers were generated, and highly informative SNP and SilicoDArT markers were selected and used for diversity analysis and the development of a representative subset containing 20% of the collection. 

### 3.2. Mapping Sesbania sesban DArTSeq markers onto the Reference Genomes of Closely Related Legume Species

In an effort to understand the distribution and select markers of known positions in the genome for downstream analyses, we explored the literature on the sequence information of legume species and reference genomes that were available in the public domain for a few legumes. We used the closely related legume reference genomes to map the *Sesbania sesban* markers generated in this study. We also used the *Sesbania sesban* transcriptome sequences available in the public domain [12]. Approximately 18% of the markers (SNP) mapped onto the transcriptome. However, the transcriptome sequences were at the scaffold level, making it difficult to select genome-wide representative markers for further analysis. Taxonomically, *Sesbania sesban* belongs to the clade Hologalegina in the subfamily Papilionoideae of the leguminosae family [19,20]. The publicly available genomes of the legumes with a similar basic chromosome number to *Sesbania sesban* such as *Medicago truncatula*, *Lotus japonicus* and *Pisum sativum* were selected to align the markers. However, only a small proportion of the generated markers (3.29–4.39% for SNPs and 1.43–2.17% for SilicoDArT) were able to be mapped onto the reference genomes. 

The poor mapping of markers onto the reference genomes of the closely related species presented a challenge to select genome-wide representative markers for the genetic studies. Similar challenges have been observed in other forage species where the reference genomes of closely related species were used [21,22,23]. In the future, we believe that the development of a reference genome for this widely grown multipurpose forage crop will strengthen the genomic tools available to support the management and improvement of germplasm, to enhance its contribution to sustainable livestock production and to support environmental management. 

### 3.3. Genetic Diversity and Population Structure in the Collection

The diversity in the collection was partitioned into ‘between’ and ‘within’ accessions, and the result revealed that a large proportion of the total variation was contributed by the ‘within’ accessions diversity. Similarly, the within clusters’ and subpopulations’ variation also contributed a larger proportion of the total variation in the collection. This is in line with the expectation for cross-pollinated species. A similar result was reported in rye (*Secale cereale* L.), a cross-pollinated cereal grown in the temperate zone [24]. Variation between accessions was also significant (Phi = 0.473, *p*-value = 0.001 for SNP and Phi = 0.326 *p*-value = 0.001 for SilicoDArT). This is also supported by a high pairwise Fst value (mean = 0.344) demonstrating the existence of genetic differentiation between the accessions. *Sesbania sesban* is a largely cross-pollinated species [2], and this reproduction mode contributes to the diversity enrichment through recombination and segregation of alleles attributing to new genotypes in the population or new allele combinations in the genome. Besides the reproduction mode, during the exploration it could be possible that seeds were collected from multiple plants to constitute an accession, leading to the higher within accession variation (Jean Hanson, former forage genebank manager, personal communication). Moreover, the possibility of mixtures cannot be ruled out in the process of regeneration in the field due to cross-pollination that contributes to the within accession variation. 

The genetic diversity analysis revealed the presence of four main clusters in the collection, with significant genetic variation between the clusters. This shows the rich genetic variation in the collection. The analysis of molecular variance (AMOVA) showed a significant difference between the identified clusters, with up to 35.72% variation between the clusters. In the case of subpopulations identified using Structure analysis, within subpopulations’ variation contributed almost all the total diversity (98.95%) in the collection, indicating the major contribution of between accessions’ variation to the total diversity in the collection, with limited stratification into subpopulation. The rich diversity and the clusters observed could be attributed to the outcrossing nature of the crop and the possible admixture of seeds during exploration. The rich genetic variation reported here is in line with the presence of considerable variation in soluble phenolic and insoluble proanthocyanin compounds in the *Sesbania sesban* collection, described elsewhere [2]. Together with morphological and chemical traits, the diversity in the species collection could be exploited for the development of cultivars through hzybrization with closely related species with better feed quality for livestock production and through developing improved high-yielding varieties with better tolerance to stresses such as saline, soil acidity and aluminum toxicity. Thus, the molecular information could be used to tag the different species in the potential hybrids and to select genotypes for improvement programs. The collection contained germplasm from different geographical origins; however, no statistically significant correlation was observed between the geographical and genetic distances. This shows the lack of genetic differentiation by geographical origin in the *Sesbania sesban* collection. An earlier study using low-density markers also showed the lack of direct relationship between genetic similarity/dissimilarity and geographic location (distance) for 11 *Sesbania sesban* populations [9]. The diversity analysis also revealed a large proportion of the total variation contributed by the within populations’ (by origin) variation compared to the between populations’ variation. The current result suggests the existence of variation within populations from each geographic origin and the need to systematically target the niche variation within the populations of different geographical origins.

### 3.4. Subset Development 

We established a representative subset containing 20% of the *Sesbania sesban* collection maintained in the ILRI genebank, using the generated markers. After calculating identity-by-descent, a representative sample per accession (with pairwise kinship value ≥0.30) was selected, and a subset containing 34 accessions was identified. Over 96–98% of the total variation was contributed by within groups’ variation, indicating the representativeness of the identified subset. 

The subset contained germplasm from a range of African countries, representing germplasm from low, medium and high-altitude areas, indicating its wide climatic representation. We believe the inclusiveness of germplasm from different ranges of altitudes in the subset complements the diversity niche, representing the diverse agro-ecologies occupied by the species. 

### 3.5. Gap Analysis and Identification of Niche Diversity to Broaden the Genetic Basis of the Collection

The observed large within accessions’ and populations’ contribution to the total diversity could have implications for broadening the genetic basis of the collection; essentially, whether to collect germplasm from new geographical areas and/or to focus on crossing genotypes within the current existing collection in the genebank(s). From a conservation and management perspective, maintaining a small-sized collection, e.g., a representative subset in terms of diversity, would be more feasible as maintaining a large collection is more expensive in terms of time, space and resources. The representative subset should contain germplasm from diverse agro-ecologies and genetic backgrounds globally. However, the observed significant genetic variation among populations of different geographical origins suggests the need for gap analysis and identification of unique genotypes from the agro-ecologies where the crop is native and/or already naturalised. This is also evidenced from the geographical representation of the collection as most of the germplasm came from a few countries in Africa. *Sesbania sesban* is native to many countries in Africa and Asia [2]. Two-thirds of the ILRI collection is represented by germplasm from four African countries (Tanzania = 66 accessions, Ethiopia = 26 accessions, Malawi = 12 accessions and Kenya = 10 accessions). In addition, eleven of the 25 countries of origin are represented by one accession each. This indicates the gap in the geographical representation of the collection and the need for a strategic approach to acquire niche diversity to broaden the genetic basis to ensure the global representativeness of the collection conserved in the genebank. Hence, the results from this study could be used to guide a gap analysis towards identification of uncaptured niche diversity in the germplasm of *Sesbania sesban*. 

## 4. Materials and Methods

### 4.1. Plant Materials

**Figure 9:**
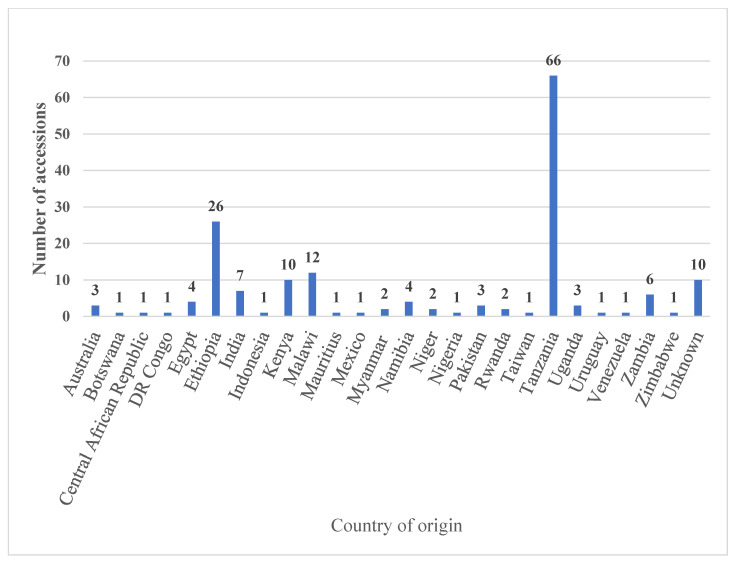
*Sesbania sesban* accessions studied by their country of origin.

One hundred and seventy-one accessions of *Sesbania sesban*, collected from different parts of the world, were used in this study (Figure 9, Supplementary Table S1). Seeds were germinated on moist germination paper in an incubator set at 25 °C. The germinated seedlings were transferred to pots filled with a sterilized (at 180 °C for 24 h) medium containing sand, manure, and forest soil in the ratio of 1:2:3 and grown in a greenhouse until big enough for the collection of leaf samples. 

### 4.2. DNA Extraction and Genotyping

Leaf samples were collected from multiple individual plants per accession and freeze-dried (Model: Labocon lfd-101). Freeze-dried leaf samples were ground to a fine powder using a TissueLyser II (Cat. No./ID: 85300), and DNA was extracted from the powdered leaf samples using a DNeasy Plant Mini kit (Cat No./ID:69106) according to the manufacturer’s instructions. The DNA quantity and quality were checked using a DeNovix spectrophotometer (mode: DS-11^+^). DNA samples were diluted to a concentration of 50–100 ng/µL, and 30 µL of the diluted samples were aliquoted into fully skirted 96-well plates. Finally, the samples were packed and shipped to SEQART Africa (previously known as Integrated Genotyping Service and Support, IGSS) in Kenya for genotyping. 

Genotyping-by-sequencing (GBS) was performed on the DArTSeq platform, and DArTSeq markers were generated as described elsewhere [13]. The generated markers were aligned with reference genomes of *Medicago truncatula* [14], *Lotus japonicus* [15] and *Pisum sativum* [16]. The transcriptome sequence of *Sesbania sesban* [12] was also used to map the generated markers. Genome-wide distribution of the generated markers was visualised using the R package Synbreed [25]. 

### 4.3. Data Analysis 

The genotyping data were analysed using different R statistical software packages (https://www.r-project.org/, accessed on 16 January 2019). The percentage of missing data, allele frequency and polymorphic information content (PIC) were calculated using a locally written script in R. The PIC values were calculated using the formula PIC = 1 − ∑X*_i_*^2^_,_ where X*_i_* is the frequency of *i*th allele of the marker [26]. Marker fragment lengths were summarised using the R package stringr [27]. Markers were filtered for missing data percentage (≤20%) and informativeness (PIC ≥ 0.2). 

Analysis of molecular variance (AMOVA) was used to partition the total genetic variation into ‘between’ and ‘within’ accessions using the R package poppr [28]. Accessions’ pairwise Fst based on two methods [29,30] was calculated using the *snpgdsFst*() function of the R package SNPRelate [18]. Linkage disequilibrium-based pruning of the SNP markers was carried out using the snpgdsLDpruning() function of the R package SNPRelate using the default settings, except for LD threshold (0.5). The snpgdsIBDMLE() function of R package SNPRelate was then used to calculate the identity-by-descent based on the pruned set of SNP markers using the maximum likelihood method. All individuals with a kinship value ≥ 0.3 were retained for diversity analysis. To study the genetic relatedness between the accessions, Euclidean genetic distances were calculated using the *dist()* function in R. Mantel correlation coefficient was calculated using the R package vegan [31] to assess the relationship between the genetic distances from the two marker types as well as between the genetic and geographical distances. The Euclidean genetic distance was converted to a hierarchical cluster (hclust object) using the *hclust()* function in R which was then converted into a dendrogram using the R package dendextend [32]. The *fviz_cluster()* function of the R package factoextra [33] was used to visualize the cluster plots of the accessions. The dendrogram (phylogenetic tree) was visualised using the *plot()* function in R. The cophenetic correlation coefficient of the dendrograms was calculated using the *cor-cophenetic()* function of the R package dendextend [32]. The optimal number of clusters was determined using the *find.clusters()* function of the R package adegenet [34]. The discriminant analysis of principal components (DAPC) function of R package adegenet [34] was used to infer the cluster membership probability and assign individual samples into the different clusters. The cluster membership and assignment of the samples were visualised using the *compoplot()* and *assignplot()* functions of the R package adegenet [34]. 

Population structure was analysed using the admixture model in STRUCTURE [35,36], and the probability of two to ten subpopulations (K) was estimated using the admixture model, 100,000 Markov Chain Monte Carlo (MCMC) repetitions and a 100,000 burn-in period. The result of the run was uploaded online to the software “STRUCTURE HARVESTER” [37], and the optimal number of subpopulations was determined using the Evanno delta K method [38]. 

The SNP markers were used to develop a subset containing 20% of the collection, representing the maximum amount of genetic diversity contained in the collection. A representative sample per accession was selected based on pairwise kinship value ≥ 0.30 and used for subset development using the R package CoreHunter v.3.1 [39]. The diversity and representativeness of the developed subset was assessed using AMOVA.

## 5. Conclusions

*Sesbania sesban* is a multipurpose legume tree with significant roles in crop–livestock-based production systems. Understanding the germplasm resources of *Sesbania sesban* maintained in the genebank is important for sustainable conservation and improvement of the species and to promote the wide use of potential genotypes to enhance the contribution of livestock to sustainable development through increased production of improved forages. In this study, we studied a *Sesbania sesban* collection held in the ILRI forage genebank and generated a large set of genotyping data using the DArTSeq platform. Diversity analysis using a subset of informative markers revealed the presence of rich genetic diversity in the collection, with little or no evidence of genetic variation according to the geographical origin of the germplasm. The genetic diversity analysis also revealed a large proportion of the variation contributed by the ‘within’ compared to the ‘between’ accessions’ and populations’ variability. We also developed a genetically representative subset containing germplasm from diverse origins. The generated genetic diversity information and the established subset could promote further research and greater use of *Sesbania sesban* germplasm. Phenotypic assessment of the representative subset for agronomical and morphological traits across agro-ecologies will help in the identification of best-bet accessions for improved performance and value in specific ecologies or across different growing environments. The informative markers could be used to guide gap analysis to capture niche diversity from geographic areas not or less represented in the collection as well as in the future endeavors of marker-assisted identification of stress-tolerant adaptable genotypes to different agro-ecologies and soil characteristics. The lack of a reference genome for the species has limited our capability to select genome-wide markers for downstream analysis. Hence, development of a reference genome should be considered in the future to accelerate breeding and selection efforts in this important multipurpose legume tree. In general, the generated information could play a vital role in the future efforts of developing and promoting climate-resilient varieties of this forage legume to support the production of forages and forage-based agroforestry/landscape management practices.

## Figures and Tables

**Table 1:** Number of markers mapped onto reference genomes.

Reference Genomes	Number and Percentage of Markers Mapped			
	SNP (*N* = 84,673)		SilicoDArT (*N* = 60,626)	
	Number	Percentage	Number	Percentage
Sesbania sesban *	15,234	17.99	5483	9.04
*Lotus japonicus*	3724	4.39	1144	1.89
*Pisum sativum*	3344	3.95	1319	2.17
*Medicago truncatula*	2790	3.29	864	1.43

* Transcriptome sequence.

**Table 2:** AMOVA result showing the contribution of ‘between’ and ‘within’ accessions diversity to the total diversity in the collection.

Marker Type	Source of Variation	Degrees of Freedom	Sum of Squares	Mean Sum of Squares	Sigma	Variation (%)	Phi	*p*-Value
SNP *	Between accessions	167	1,143,711	6848.57	469.73	47.27	0.473	0.001
	Within accessions	2098	1,099,140	523.90	523.90	52.73		
	Total	2265	2,242,851	990.22	993.63			
SilicoDArT **	Between accessions	167	3,669,694	21,974.22	1415.08	32.64		
	Within accessions	2098	6,128,065	2920.91	2920.91	67.36	0.326	0.001
	Total	2265	9,797,759	4325.72	4335.99	100.00		

* 7587 SNP markers; ** 15,031 SilicoDArT markers.

**Table 3:** AMOVA result for clusters inferred by hierarchical clustering.

Method	Marker	Source of Variation	Degrees of Freedom	Sum of Squares	Mean Sum of Squares	Sigma	Variation (%)	Phi	*p*-Value
Hierarchical clustering	SNP	Between clusters	3	658,336.00	219,445.32	389.16	35.72	0.357	0.001
		Within clusters	2262	1,584,176.00	700.34	700.34	64.28		
		Total	2265	2,242,512.00	990.071	1089.50	100.00		
Structure analysis	SNP	Between clusters	3	17,241.71	5747.24	10.47	1.05	0.011	0.001
		Within clusters	2262	2,225,608.85	983.91	983.91	98.95		
		Total	2265	2,242,850.56	990.22	994.38	100.00		
Hierarchical clustering	SilicoDArT	Between clusters	3	1,988,965.00	662,988.26	1251.49	26.61	0.266	0.001
		Within clusters	2262	7,808,795.00	3452.16	3452.16	73.39		
		Total	2265	9,797,759.00	4325.72	4703.65	100.00		

**Table 4:** AMOVA result for the populations according to the accessions’ countries of origin.

Marker	Source of Variation	Degrees of Freedom	Sum of Squares	Mean Sum of Squares	Sigma	Variation (%)	Phi	*p*-Value
SNP	Between populations	25	501,209.8	20,048.39	256.36	24.80	0.248	0.001
	Within populations	2240	1,741,640.8	777.52	777.52	75.20		
	Total	2265	2,242,850.6	990.22	1033.88	100.00		
SilicoDArT	Between populations	25	1,506,995.0	60,279.82	752.66	16.90	0.169	0.001
	Within populations	2240	8,290,764.0	3701.23	3701.23	83.10		
	Total	2265	9,797,759.0	4325.72	4453.90	100.00		

**Table 5:** List of accessions with DOI identifiers and origin contained in the subset, developed using SNP markers.

DOI	Accession Code	Country of Origin	Latitude	Longitude	Elevation
10.18730/G7QE=	920	Tanzania	−1.3821	34.2823	
10.18730/FQPKF	1180	Tanzania	−6.3483	36.4813	900
10.18730/FQT5J	1191	Tanzania	−8.8413	34.1676	1050
10.18730/FQTV3	1193	Tanzania	−8.8324	33.8688	1060
10.18730/FQVGR	1195	Tanzania	−9.1166	32.9237	1550
10.18730/FR21B	1215	Tanzania	−4.9191	29.6036	780
10.18730/FR3V*	1221	Tanzania	−4.0411	30.5473	1120
10.18730/FR8EZ	1237	Tanzania	−2.641	30.994	1280
10.18730/FRAY0	1246	Tanzania	−2.6575	32.6592	1100
10.18730/FRFYC	1262	Tanzania	−3.787	35.862	920
10.18730/FRQDX	1286	Tanzania	−4.65	38.0833	400
10.18730/FRRDR	1289	Tanzania	−4.9333	38.3	385
10.18730/FYRK*	2000	Ethiopia	8.35	39.33	1750
10.18730/FZBC4	2055	Ethiopia	10.9833	36.4333	1700
10.18730/FZC2T	2057	Ethiopia	11	36.4	1740
10.18730/G7HPU	8740	Ethiopia	6.4167	37.2	1120
10.18730/FPJQE	10521	Ethiopia	6.8333	37.7667	1925
10.18730/FPNT2	10639	Ethiopia	7.75	36.5667	1640
10.18730/FRXRA	13144	Kenya	0.5833	34.5667	1450
10.18730/FTAXC	15020	Kenya			
10.18730/FTAYD	15021	Uganda			
10.18730/FTC6G	15077	India			
10.18730/FTMJS	15364	Kenya	−0.7333	36.4333	1890
10.18730/FVJY=	16514	Central African Republic	8.4833	21.2167	600
10.18730/FVPB~	16626	Namibia	−17.2167	12.4167	250
10.18730/FWB6$	17313	Unknown			
10.18730/FWBKA	17326	Zambia	−15.75	26.05	1120
10.18730/FWCH3	17356	Malawi	−14.6167	35.3167	472
10.18730/FWCSB	17364	Malawi	−14.0167	33.35	1150
10.18730/FWCTC	17365	Malawi	−13.6667	34.5833	415
10.18730/FWCVD	17366	Malawi	−13.15	34.3333	474
10.18730/FWCYG	17369	Malawi	−10.4833	34.2	480
10.18730/G2N6Q	23701	Zimbabwe	−17.827	31.0514	1484
10.18730/G2P5H	23733	Mexico	27.75	−110.5	50

**Table 6:** AMOVA result between the subset and the rest of the germplasm.

Marker	Source of Variation	Degrees of Freedom	Sum of Squares	Mean Sum of Squares	Sigma	Variation (%)	Phi	*p*-Value
SNP	Between groups	1	3087.77	3087.77	38.29	3.65	0.036	0.0001
	Within groups	166	167,794.35	1010.81	1010.81	96.35		
	Total	167	170,882.13	1023.25	1049.10	100.00		
SilicoDArT	Between groups	1	7198.437	7198.44	50.08	1.11	0.011	0.0083
	Within groups	166	744,008.13	4481.98	4481.98	98.89		
	Total	167	751,206.56	4498.24	4532.06	100.00		

## Data Availability

All data generated in this study are freely available as international public goods.

## Supplementary Materials

The following supporting information can be downloaded at: https://www.mdpi.com/article/10.3390/plants12010013/s1. Figure S1: Genome-wide distribution and density of DArTSeq markers on the selected reference genomes; Figure S2: Heatmap showing accession pairwise Fst value based on the W&H02 method; Figure S3: Heatmap showing population (by origin) pairwise Fst value based on the W&H02 method; Table S1: Passport data of the *Sesbania sesban* collection.
