# Supplementary material for: Genetic Diversity, Population Structure and Subset Development in a Sesbania sesban Collection
Source: Plants (Basel). 2022 Dec 20;12(1):13. doi: 10.3390/plants12010013 (PMC9823922; doi:10.3390/plants12010013)
Supplement: Supplementary file 1 [file plants-12-00013-s001.zip › Supplemenatary Figures.docx]

**Figure S1.** Genome‐wide distribution and density of DArTSeq markers on the selected reference genomes. The chromosome code is shown on the x-axis and the number of markers mapped per chromosome are shown on the y-axis. The markers that were not mapped onto a specific chromosome or chloroplast DNA (CpDNA) or Mitochondria DNA(MtDNA) are indicated as Unknown.
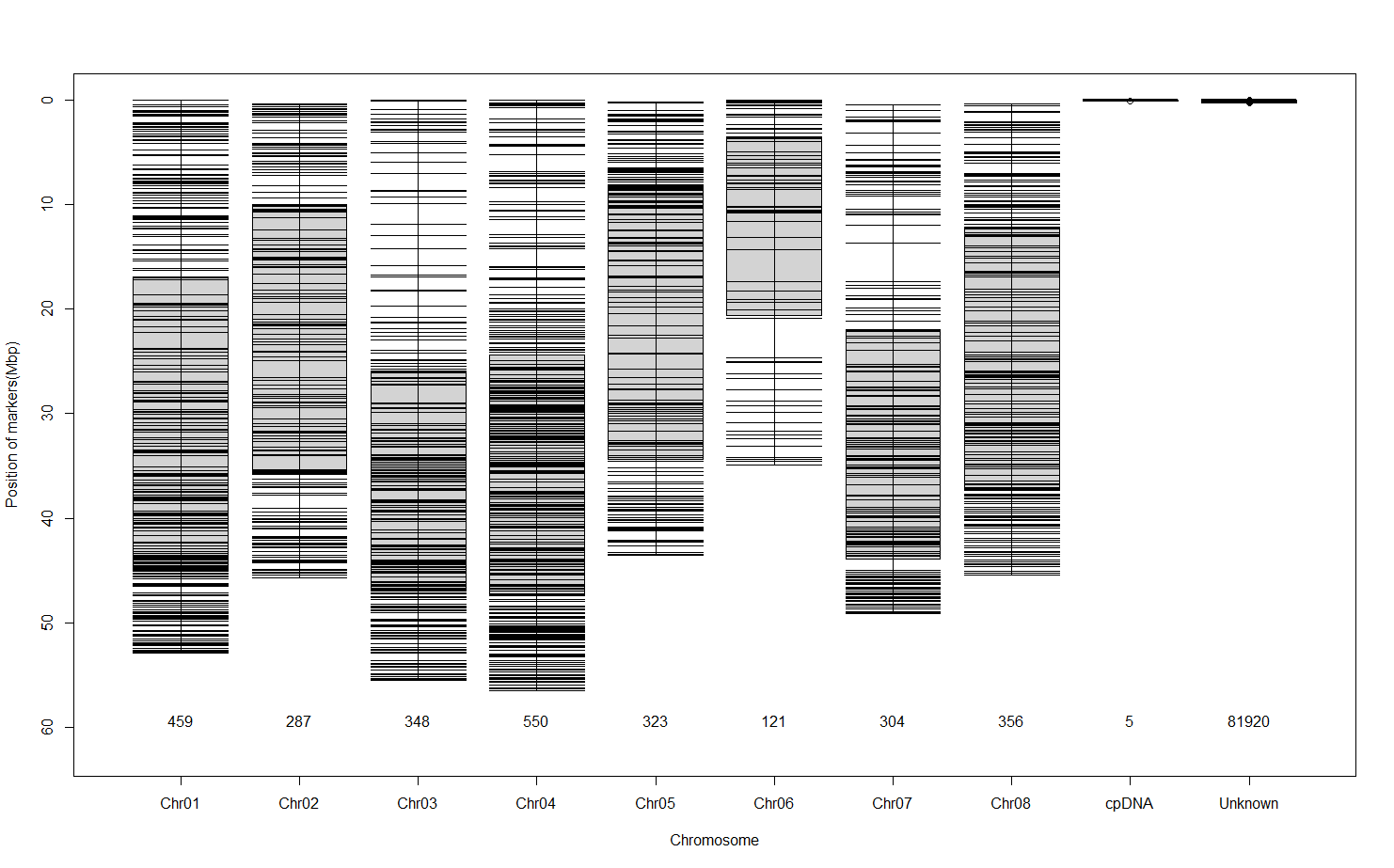


1. SNP markers mapped onto the *Medicago truncatula* genome.


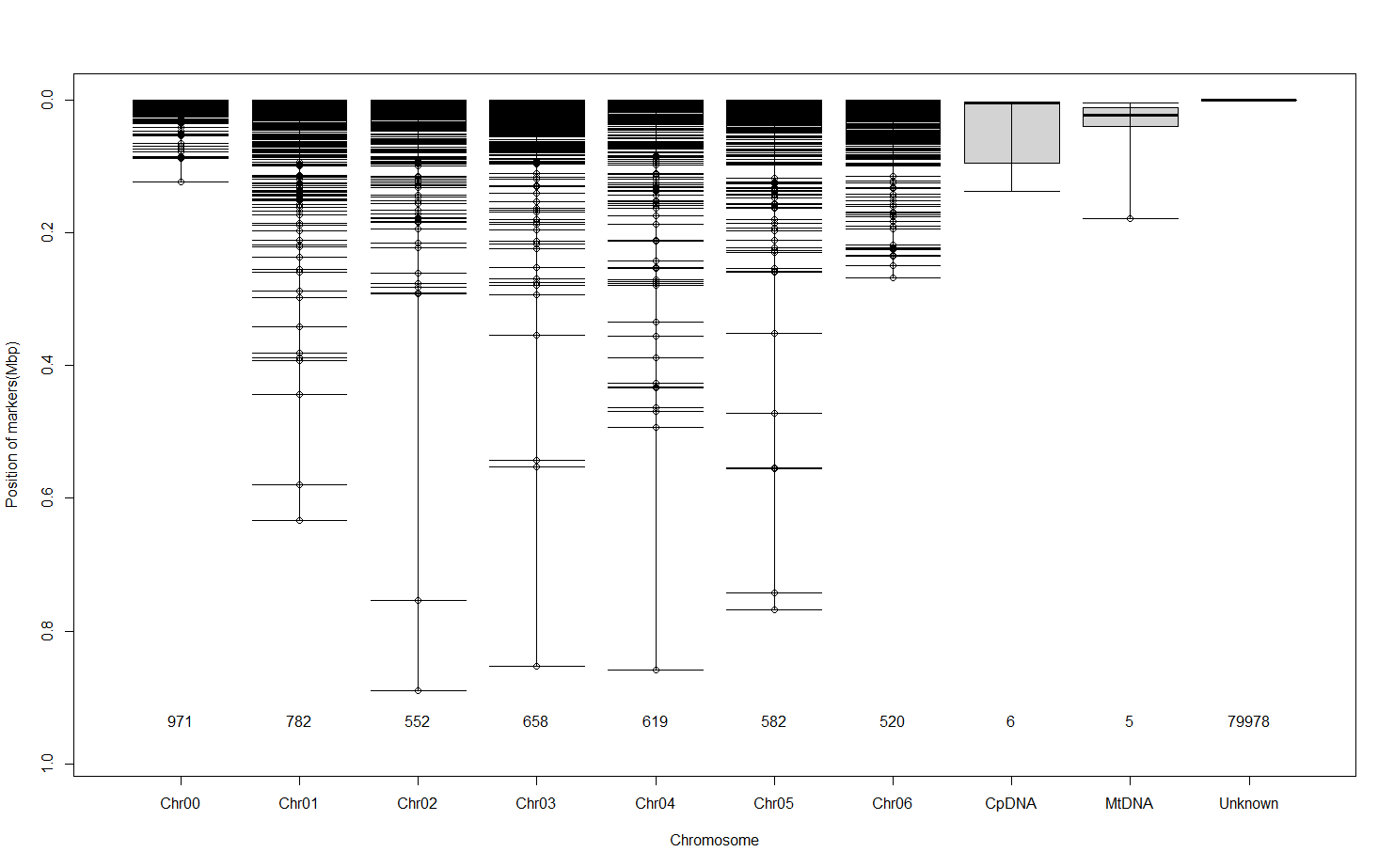


1. SNP markers mapped onto the *Lotus japonicus* genome.


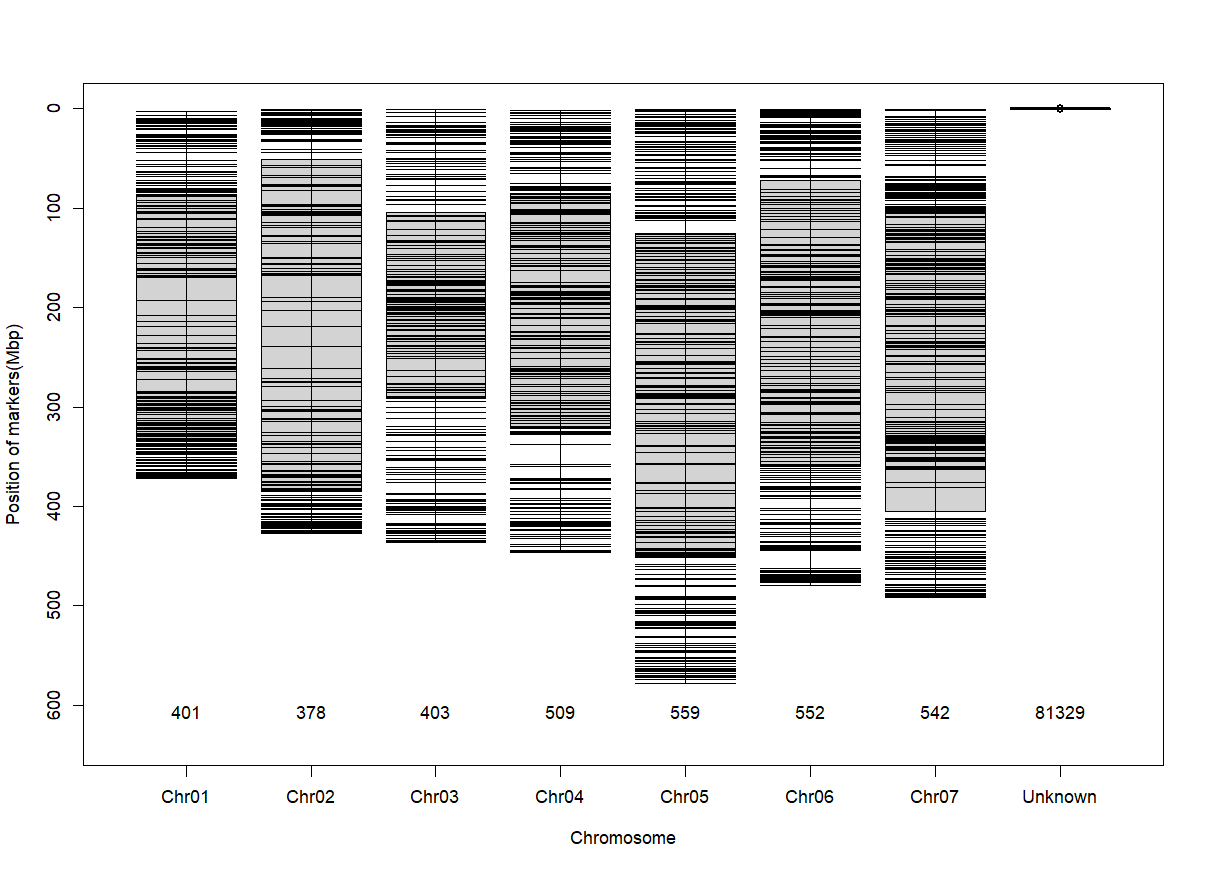


1. SNP markers mapped onto the *Pisum sativum* genome.


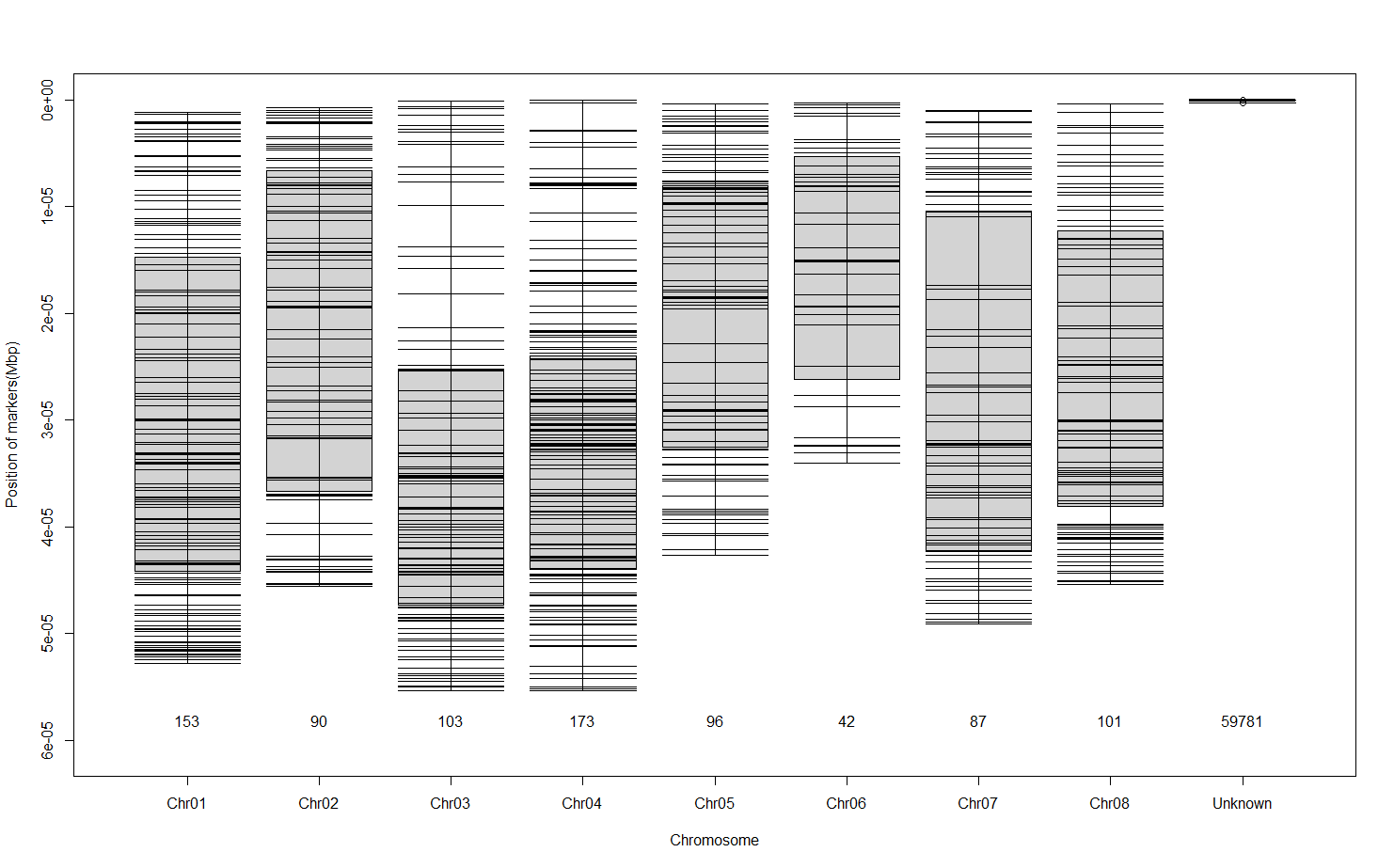


1. SilicoDArT markers mapped onto the *Medicago truncatula* genome.


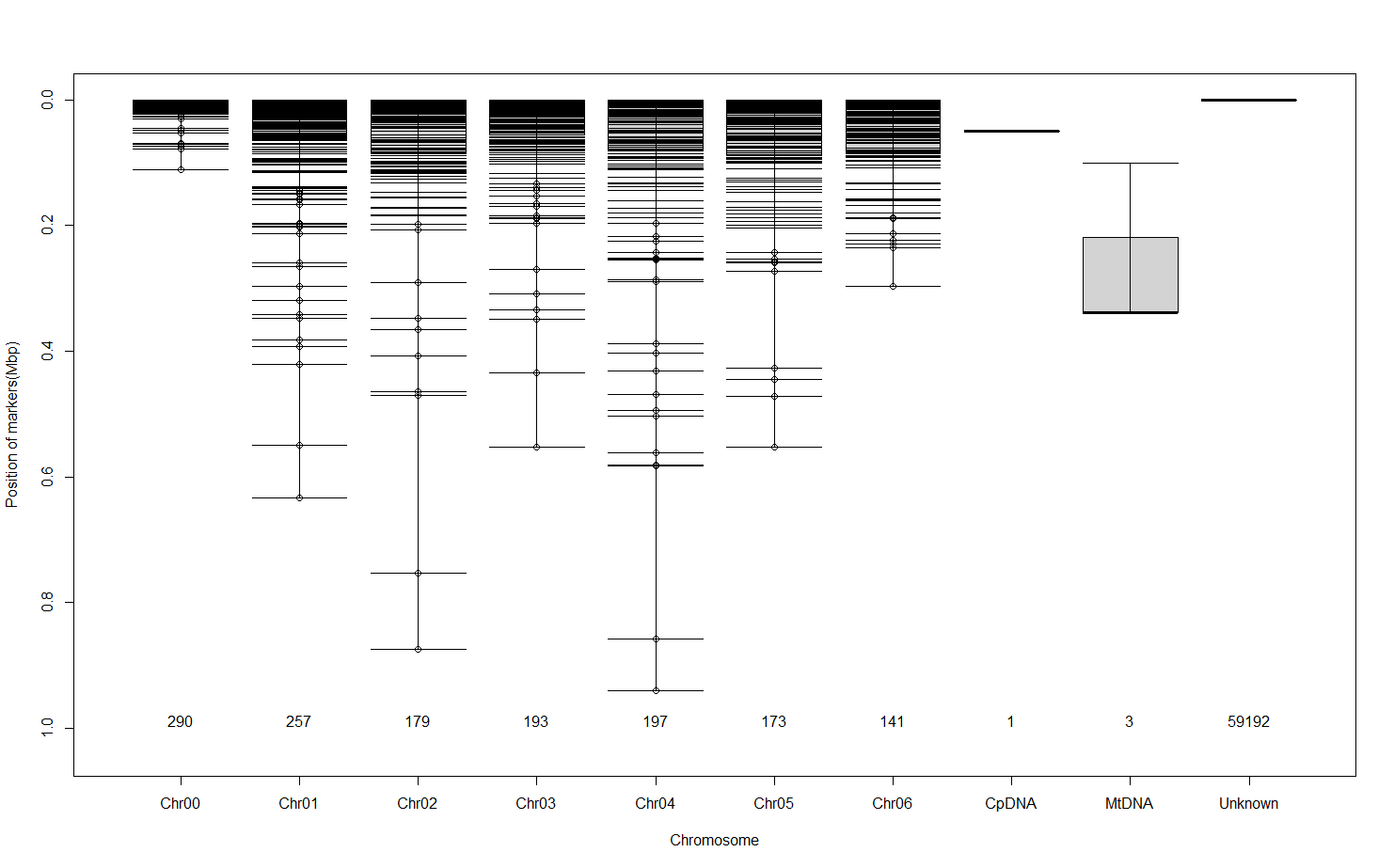


1. SilicoDArT markers mapped onto the *Lotus japonicus* genome


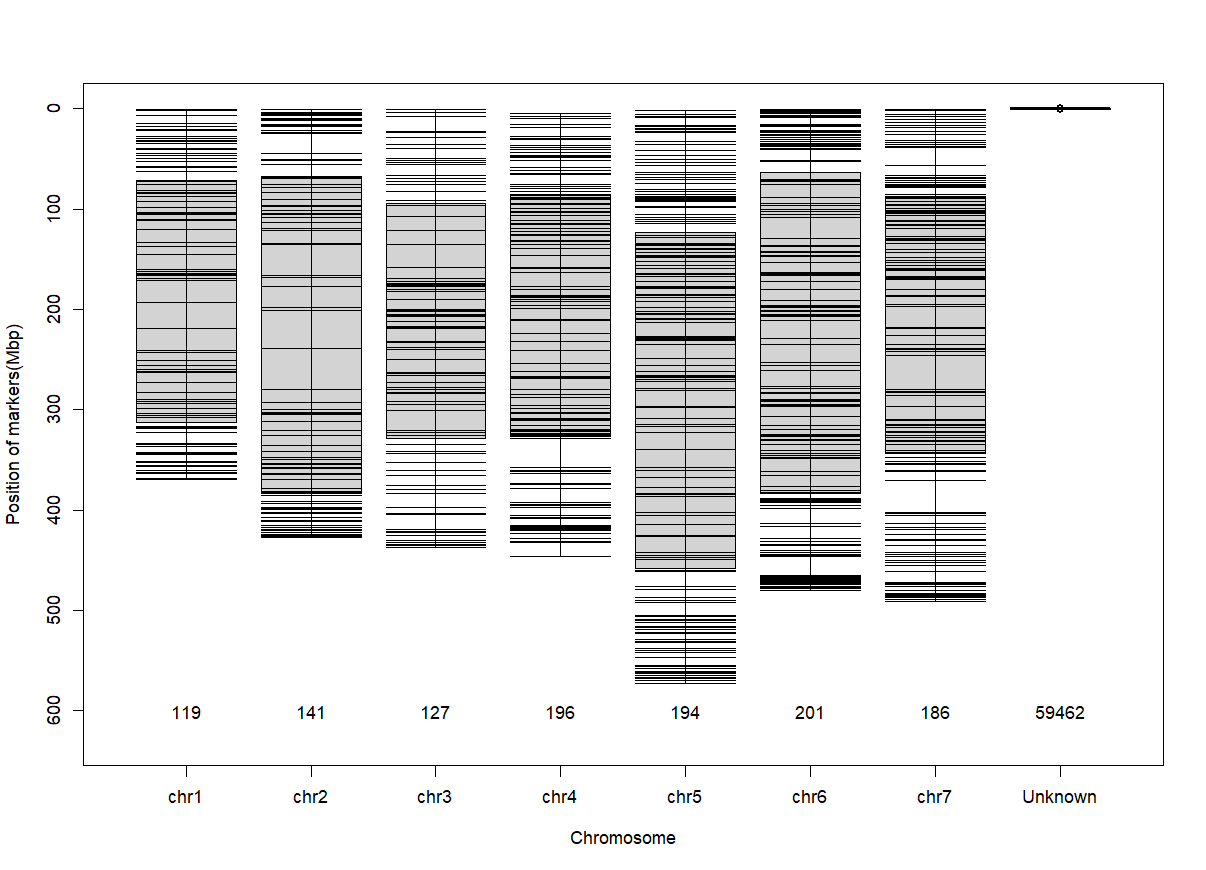


1. SilicoDArT markers mapped onto the *Pisum sativum* genome

Figure S2. Heatmap showing accession pairwise Fst value based on the W&H02 method


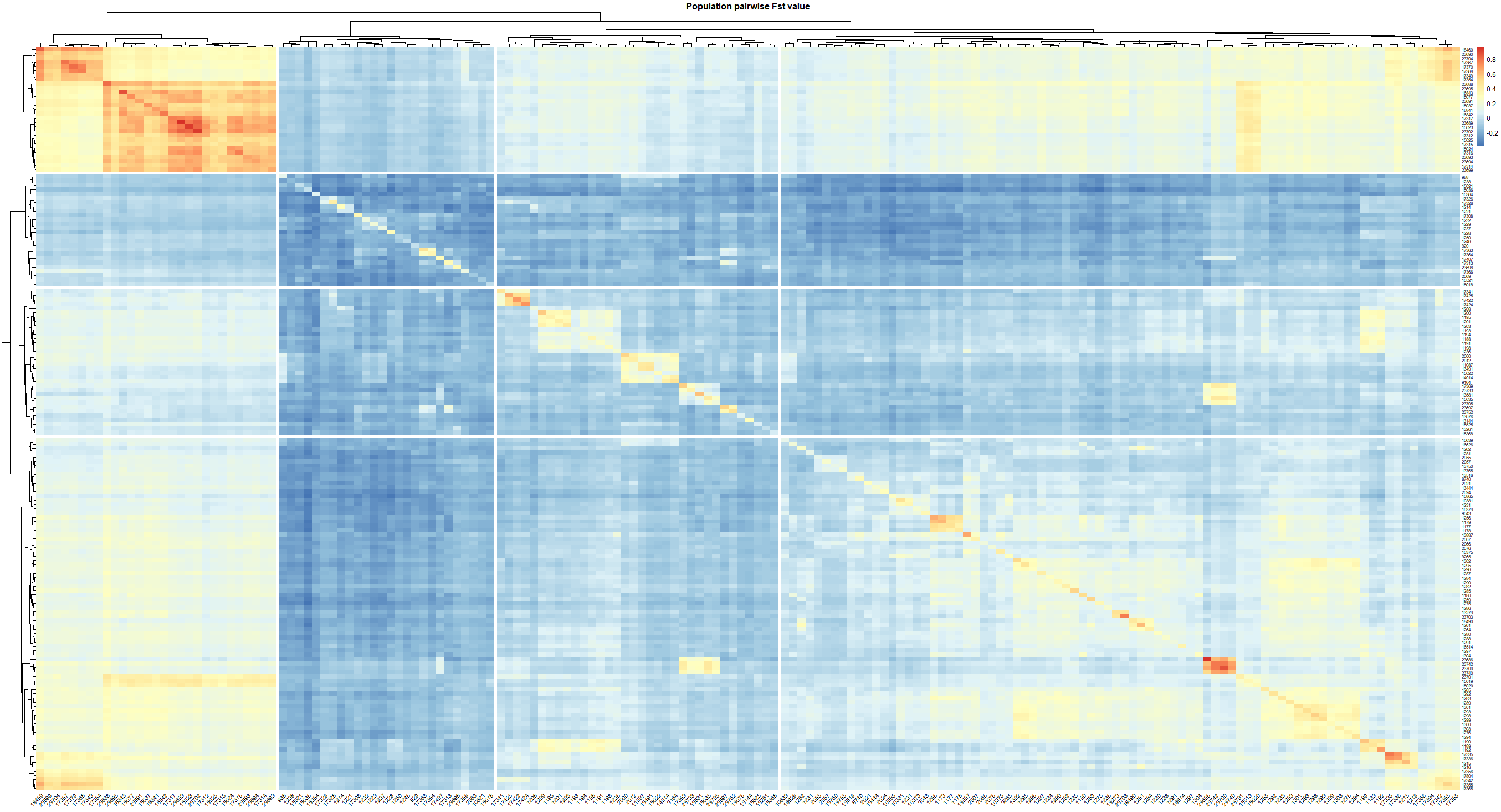


Figure S3. Heatmap showing population (by origin) pairwise Fst value based on the W&H02 method


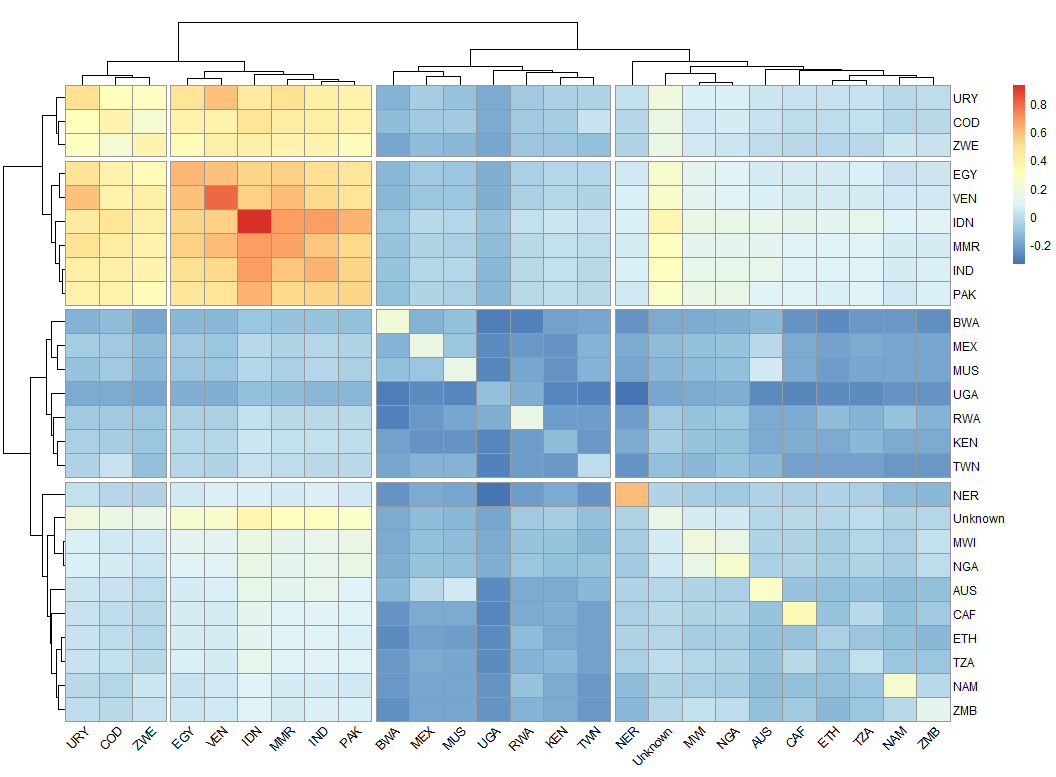
Aus: Australia; BWA: Botswana; CAF: Central African Republic; COD: DR Congo; EGY: Egypt; ETH: Ethiopia; IND: India; IDN: Indonesia; KEN: Kenya; MWI: Malawi; MUS: Mauritius; MEX: Mexico; MMR: Myanmar; NAM: Namibia; NER: Niger; NGA: Nigeria; PAK: Pakistan; RWA: Rwanda; TWN: Taiwan; TZA: Tanzania; UGA: Uganda; URY: Uruguay; VEN: Venezuela; ZMB: Zambia; ZWE: Zimbabwe
